# Liquid biopsy epigenomic profiling for cancer subtyping

**DOI:** 10.1038/s41591-023-02605-z

**Published:** 2023-10-21

**Authors:** Sylvan C. Baca, Ji-Heui Seo, Matthew P. Davidsohn, Brad Fortunato, Karl Semaan, Shahabbedin Sotudian, Gitanjali Lakshminarayanan, Miklos Diossy, Xintao Qiu, Talal El Zarif, Hunter Savignano, John Canniff, Ikenna Madueke, Renee Maria Saliby, Ziwei Zhang, Rong Li, Yijia Jiang, Len Taing, Mark Awad, Cindy H. Chau, James A. DeCaprio, William D. Figg, Tim F. Greten, Aaron N. Hata, F. Stephen Hodi, Melissa E. Hughes, Keith L. Ligon, Nancy Lin, Kimmie Ng, Matthew G. Oser, Catherine Meador, Heather A. Parsons, Mark M. Pomerantz, Arun Rajan, Jerome Ritz, Manisha Thakuria, Sara M. Tolaney, Patrick Y. Wen, Henry Long, Jacob E. Berchuck, Zoltan Szallasi, Toni K. Choueiri, Matthew L. Freedman

**Affiliations:** 1 Department of Medical Oncology, Dana-Farber Cancer Institute, Boston, MA USA; 2 Center for Functional Cancer Epigenetics, Dana-Farber Cancer Institute, Boston, MA USA; 3 Eli and Edythe L. Broad Institute, Cambridge, MA USA; 4 Computational Health Informatics Program, Boston Children’s Hospital, Boston, MA USA; 5 Lowe Center for Thoracic Oncology, Dana-Farber Cancer Institute, Harvard Medical School, Boston, MA USA; 6 Molecular Pharmacology Section, Genitourinary Malignancies Branch, Center for Cancer Research, National Cancer Institute, National Institutes of Health, Bethesda, MD USA; 7 Department of Medicine, Brigham and Women’s Hospital, Harvard Medical School, Boston, MA USA; 8 Liver Cancer Program, Center for Cancer Research, National Cancer Institute, National Institutes of Health, Bethesda, MD USA; 9 Massachusetts General Hospital Cancer Center, Boston, MA USA; 10 Department of Medicine, Massachusetts General Hospital and Harvard Medical School, Boston, MA USA; 11 Department of Pathology, Dana-Farber Cancer Institute, Boston, MA USA; 12 Thoracic and Gastrointestinal Malignancies Branch, Center for Cancer Research, National Cancer Institute, National Institute of Health, Bethesda, MD USA; 13 Department of Dermatology, Brigham and Women’s Hospital, Harvard Medical School, Boston, MA USA; 14 Center for Cutaneous Oncology, Dana-Farber/Brigham and Women’s Cancer Center, Boston, MA USA; 15 Center for Neuro-Oncology, Dana-Farber Cancer Institute, Boston, MA USA; 16 Department of Neurology, Brigham and Women’s Hospital and Harvard Medical School, Boston, MA USA; 17 Danish Cancer Institute, Copenhagen, Denmark; 18 Department of Bioinformatics and Department of Pathology, Forensic and Insurance Medicine, Semmelweis University, Budapest, Hungary

**Keywords:** Tumour biomarkers, Predictive markers, Biomarkers, Translational research, Epigenomics

## Abstract

Although circulating tumor DNA (ctDNA) assays are increasingly used to inform clinical decisions in cancer care, they have limited ability to identify the transcriptional programs that govern cancer phenotypes and their dynamic changes during the course of disease. To address these limitations, we developed a method for comprehensive epigenomic profiling of cancer from 1 ml of patient plasma. Using an immunoprecipitation-based approach targeting histone modifications and DNA methylation, we measured 1,268 epigenomic profiles in plasma from 433 individuals with one of 15 cancers. Our assay provided a robust proxy for transcriptional activity, allowing us to infer the expression levels of diagnostic markers and drug targets, measure the activity of therapeutically targetable transcription factors and detect epigenetic mechanisms of resistance. This proof-of-concept study in advanced cancers shows how plasma epigenomic profiling has the potential to unlock clinically actionable information that is currently accessible only via direct tissue sampling.

## Abstract (web summary)

In a large multi-cancer cohort, a single liquid biopsy assay enables the detection of four epigenomic modifications, allowing the monitoring of expression of potential drug targets and resistance genes.

## Main

Circulating tumor DNA (ctDNA) analysis is gaining traction in clinical oncology as a minimally invasive means to detect targetable alterations and monitor cancer recurrence or persistence. Most clinical ctDNA assays focus on genomic alterations, limiting their ability to detect clinically important features of cancer that are measured from tumor tissues, such as histologic subtypes and expression of key genes. To overcome this limitation, recent efforts have focused on measuring epigenomic features from ctDNA (for example, DNA methylation^1,2^) or inferring epigenomic features from nucleosome positioning^3–5^ or DNA fragmentation patterns^6^. Most recently, profiling histone modifications from circulating nucleosomes has advanced the ability to measure gene regulation from plasma^7,8^. Histone modifications provide a dynamic readout of transcriptional programs and cellular states in cancer^9^.

**Fig. 1:**
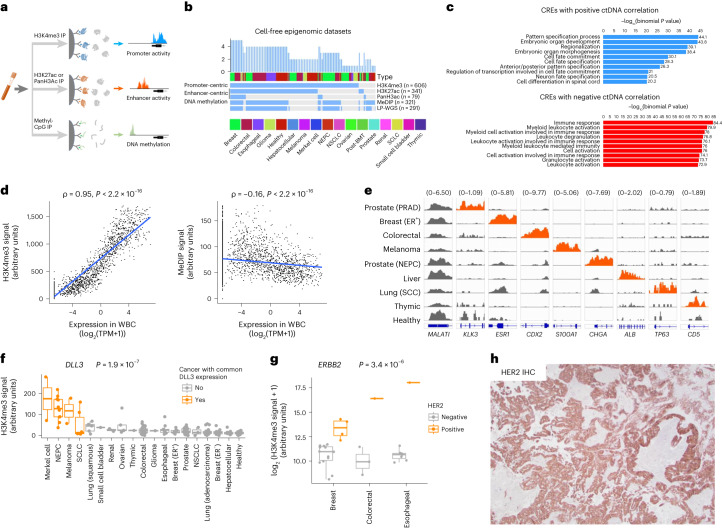
Epigenomic profiling of plasma identifies clinically actionable cancer phenotypes. **a**, Overview of the method. The indicated epigenetic marks are isolated from plasma via immunoprecipitation (IP). DNA fragments from genomic regions bearing these marks are enriched and quantified via high-throughput sequencing, providing a genome-wide assessment of promoter activity, enhancer activity and DNA methylation. **b**, Epigenomic datasets generated from plasma. post-BMT, post-bone marrow transplant. **c**, GO term enrichment for genes near REs that correlate with ctDNA content (CREs). The top 1,000 peaks by significance of correlation with ctDNA were combined for each data type (H3K4me3, H3K27ac, panH3ac and MeDIP) and jointly analyzed. **d**, Plasma signal from H3K4me3 (left) and DNA methylation (right) at gene promoters (*y* axis) in healthy donor plasma versus gene expression levels in white blood cells (WBCs; *x* axis). Each dot represents ~10 aggregated genes with similar WBC expression levels. **e**, Normalized H3K4me3 cfChIP-seq signal of diagnostic marker genes. Each row represents plasma from a patient with the indicated cancer or a healthy volunteer. Signal at each gene is scaled uniformly across plasma samples to allow for comparison. Promoter signal is shown in orange where gene expression is expected in the corresponding cancer type. **f**, Normalized H3K4me3 cfChIP-seq signal at the *DLL3* promoter stratified by cancer type for *n* = 202 biologically independent samples. Orange indicates cancer types in which the indicated gene is commonly expressed. *P* value corresponds to Wilcoxon test between cancer types with and without common expression of *DLL3*. **g**, Normalized H3K4me3 cfChIP-seq signal at the *ERBB2* promoter for *n* = 30 biologically independent samples. Samples are stratified by HER2 expression per IHC staining of tumor tissue. *P* value corresponds to Wilcoxon test between HER2^+^ and HER2^−^ cancers. **h**, IHC staining of HER2 from a brain metastasis from a patient with CRC (AMP-PL-0020-002). Scale bar, 100 μm. For **f** and **g**, only plasma samples with estimated ctDNA content >0.05 are included. For box plots, lower, middle and upper hinges indicate 25th, 50th and 75th percentiles; whiskers extend to 1.5× the interquartile ranges. All *P* values indicate two-sided tests.

Despite advances in epigenomic profiling, current approaches provide a limited view of gene regulation. To address this deficit, we developed an assay that measures multiple facets of gene regulation. Using an immunoprecipitation-based approach, our assay enriches DNA fragments from regulatory elements (REs) bearing specific epigenetic marks. We used antibodies targeting methylated DNA, H3K4me3 (a histone modification associated with promoter activity) and H3K27ac/panH3ac, histone modifications that are present at active enhancers and promoters. This strategy provides a genome-wide assessment of key regulators of gene expression: methylated DNA, active promoters and active (as opposed to poised^7,10^) enhancers (Fig. 1a). In this proof-of-concept study in cohorts of patients with advanced cancer, we demonstrate that the assay captures clinically relevant information, such as histologic subtypes, epigenetic correlates of treatment resistance and expression of predictive markers, that could potentially be used to guide therapy selection.

**Extended Data Fig. 1:**
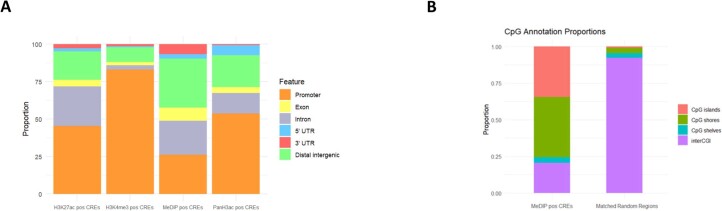
Genomic features overlapping cfChIP-seq and cfMeDIP-seq peaks. (**a**) Overlaps for the top 1,000 ctDNA-correlated regulatory elements (CREs) by significance are plotted for each assay type. (**b**) Overlap of the top 1,000 cfMeDIP-seq CREs with CpG islands, shores, and shelves. Random regions matched for chromosome and size are shown for comparison.

**Extended Data Fig. 2:**
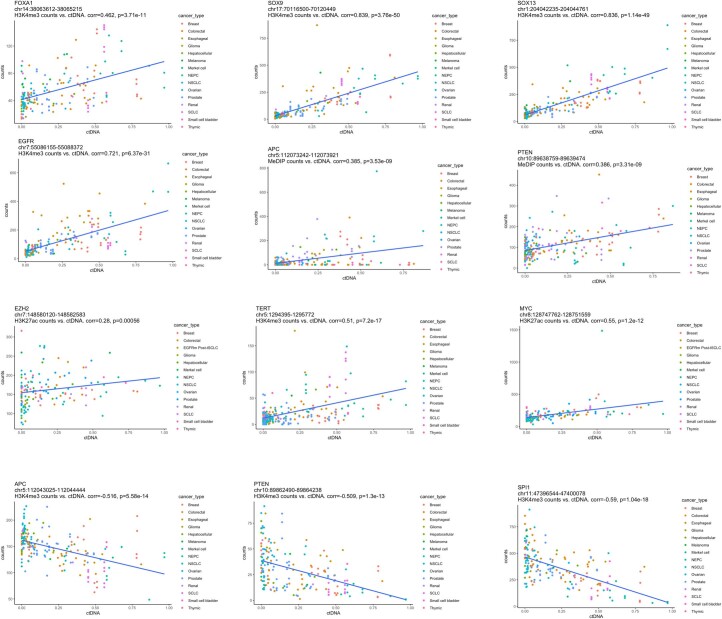
Examples of positive and negative ctDNA-correlated regulatory elements (CREs). Normalized read counts from epigenomic features correlate with ctDNA fraction at CREs. Spearman correlation coefficients and two-sided p-values are indicated.

We measured 1,268 plasma-based epigenomic profiles, including promoters, enhancers and CpG islands, from 433 individuals with one of 15 types of advanced cancer or no cancer history. (Fig. 1b, Extended Data Fig. 1 and Supplementary Table 1). We identified pan-cancer-associated REs where signal correlated with ctDNA content across plasma samples representing 15 cancer types (Methods), which we termed ctDNA-correlated REs (CREs; Extended Data Fig. 2 and Supplementary Table 2). Genes near CREs were highly enriched for functional annotations related to embryonic development and cell fate commitment (Fig. 1c), consistent with the hypothesis that cancer reactivates developmental regulatory programs^11,12^. Our CRE analysis implicated promoter activation of developmental transcription factors (TFs) (for example, *FOXA1*, *SOX9* and *SOX13*) and protooncogenes (for example, *MYC*, *EZH2* and *EGFR*), as well as repressive promoter methylation of tumor suppressor genes (for example, *APC* and *PTEN*), demonstrating that these genes can be dysregulated in cancer via epigenetic changes (Extended Data Fig. 2). CREs that negatively correlated with ctDNA were enriched for terms relating to immune function, likely reflecting RE activity from hematopoietic cells (Fig. 1c). These results indicate the biological relevance of cancer-derived epigenomic profiles from plasma.

**Extended Data Fig. 3:**
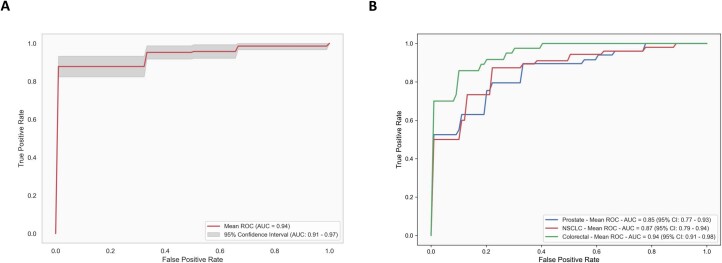
Classification of cancer plasma based on H3K4me3 cfChIP-seq profiles. (**a**) Receiver operating characteristic (ROC) curves for logistic regression-based classification of cancer plasma vs. healthy plasma, using as features the promoter H3K4me3 signal at a set of tissue-specific genes defined in the Human Protein Atlas (HPA) database^24^ (Methods). The classifier considered genes that were annotated as ‘tissue enriched’ or ‘tissue enhanced’ as well as ‘Not detected in immune cells’ in the HPA database. AUC, area under the curve. (**b**) ROC curves for classification of three cancer types with the most examples in the cohort.

**Extended Data Fig. 4:**
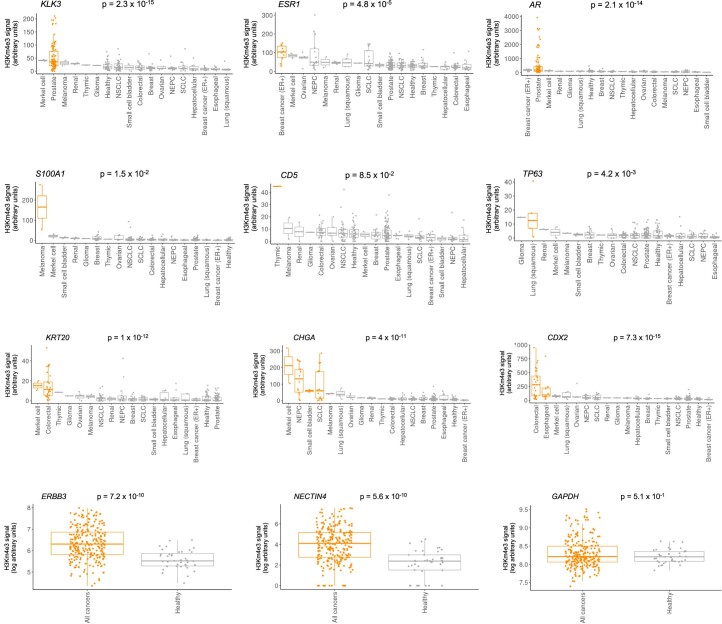
H3K4me3 cfChIP-seq signal at promoters of selected genes of interest. Promoter H3K4me3 signal is shown at selected genes across N = 202 biologically independent plasma samples stratified by cancer type. Orange indicates cancer types in which the indicated gene is expected to be expressed. Wilcoxon two-sided *p*-values are indicated for comparison of samples in which expression is expected versus all other samples. For *NECTIN4* and *ERBB3*, signal is compared between healthy volunteer plasma and cancer patient plasma because these genes are expressed across various cancer types. Signal at *GAPDH* is shown as a control. Lower, middle, and upper hinges indicate 25^th^, 50^th^, and 75^th^ percentiles; whiskers extend to 1.5 x the inter-quartile ranges (IQR).

**Extended Data Fig. 5:**
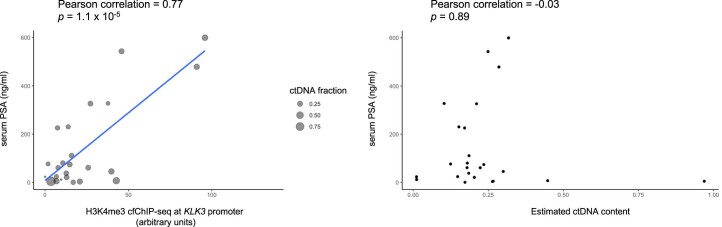
Correlation of serum PSA with H3K4me3 cfChIP-seq signal at *KLK3*. Correlation of serum PSA with ctDNA content is shown as a comparison. Pearson two-sided *p*-values are indicated.

Our assay provides a proxy for cancer gene expression from plasma. Plasma H3K4me3 signal correlated with gene expression levels measured in cells (Fig. 1d) and expression of diagnostic and predictive biomarkers in cancer. Promoter signal at lineage-enriched genes distinguished cancer types (Extended Data Fig. 3) and reflected patterns of protein expression observed in tissues by immunohistochemistry (IHC; Fig. 1e and Extended Data Fig. 4). For instance, H3K4me3 signal was enriched at the diagnostic genes *CHGA*, *CDX2* and *KRT7* in plasma from patients with neuroendocrine cancers, gastrointestinal cancers and colorectal cancer (CRC) or Merkel cell cancer, respectively (Extended Data Fig. 4). *KLK3*, which encodes the prostate cancer biomarker PSA, demonstrated elevated signal in prostate cancer plasma (*P* = 2.3 × 10^−15^; Extended Data Fig. 5) that correlated with serum PSA measurements (Pearson correlation coefficient 0.77, *P* = 1.1 × 10^−5^). *KLK3* signal did not correlate with tumor DNA fraction (Extended Data Fig. 5). This result indicates that our assay reflects variability in promoter activity at the *KLK3* locus rather than solely reflecting levels of ctDNA.

Notably, this assay measured promoter activity of genes encoding drug targets, such as *ERBB2*, *ERBB3*, *NECTIN4* and *DLL3* (Fig. 1f,g and Extended Data Fig. 4). For instance, a plasma sample from a patient with CRC demonstrated elevated signal at the *ERBB2* promoter, suggesting expression of human epidermal growth factor receptor 2 (HER2), which was confirmed subsequently by IHC of a brain metastasis biopsy (Fig. 1h). HER2 is a validated target in CRC but is not consistently assessed owing to its low prevalence (~3%) (ref. ^13^), a challenge that could be overcome by a blood-based assay.

**Fig. 2:**
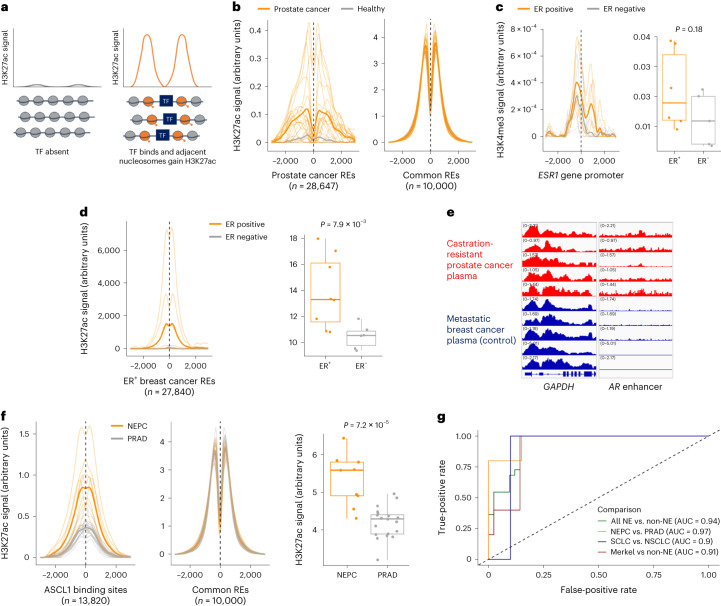
Plasma enhancer profiling enables detection of NE-diff across multiple cancers. **a**, Schematic demonstrating the measurement of enhancer activity at REs or TFBSs based on H3K27ac cfChIP-seq signal. **b**, Aggregate H3K27ac cfChIP-seq signal at REs identified by ATAC-seq in prostate tumor tissue^14^. Signal in prostate cancer plasma and healthy plasma are colored orange and gray, respectively. Dark lines show the mean signal across all samples in the indicated class. For comparison, signal at ‘common’ REs is shown, which include 10,000 REs with DNAse hypersensitivity across most or all cell types^20^ (Methods). See also Extended Data Fig. 6. **c**, Normalized H3K4me3 cfChIP-seq signal in breast cancer patient plasma at the *ESR1* gene promoter (*n* = 19 biologically independent samples). Dark lines indicate the mean signal across all samples in a class (ER^+^ or ER^−^). Box plots show AUC for cfChIP profiles. Wilcoxon test *P* values are indicated for comparison of ER^+^ versus ER^−^ breast cancer. **d**, H3K27ac cfChIP-seq signal in breast cancer patient plasma (*n* = 17 biologically independent samples) at REs with preferentially accessible chromatin in ER^+^ breast cancer^4^. Signal is aggregated across 27,840 REs for each sample. Dark lines indicate the mean signal across all samples in a class (ER^+^ or ER^−^). Box plots show AUC for the aggregate H3K27ac cfChIP profile for each sample. Wilcoxon test *P* values are indicated for comparison of ER^+^ versus ER^−^ breast cancer. **e**, H3K27ac cfChIP-seq signal at the *AR* gene enhancer in patients with castration-resistant prostate cancer. Plasma from patients with metastatic breast cancer is included as a control. **f**, Aggregated H3K27ac cfChIP-seq signal at ASCL1 binding sites for prostate cancer with and without NE-diff (NEPC and PRAD, respectively; *n* = 33 biologically independent samples). Box plots indicate AUC for the aggregate H3K27ac profile for each sample. Wilcoxon test *P* values are indicated for comparison of NEPC versus PRAD. **g**, ROC curves for distinguishing samples with NE-diff using H3K27ac cfChIP-seq signal at neuroendocrine REs. ‘AUC’ indicates area under the ROC curve for each comparison. For **a**–**c**, only plasma samples with estimated ctDNA content >0.03 are included. For all box plots, lower, middle and upper hinges indicate 25th, 50th, and 75th percentiles; whiskers extend to 1.5× the interquartile ranges. All *P* values indicate two-sided tests. NE, neuroendocrine; PRAD, prostate adenocarcinoma.

**Extended Data Fig. 6:**
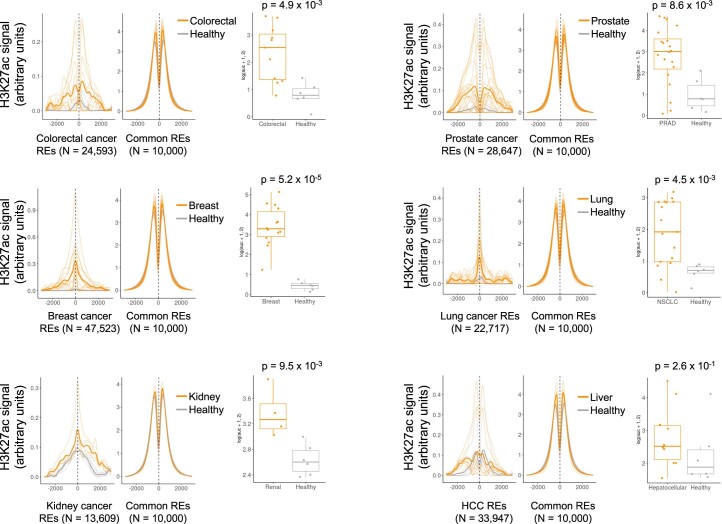
Aggregate H3K27ac cfChIP signal at regulatory elements identified by ATAC-seq in tumor tissue. Signal in cancer plasma (orange) and healthy plasma (gray) is compared at regulatory elements in the corresponding cancer type defined by ATAC-seq in TCGA tumors^14^. Dark lines show the mean signal across all samples in the indicated class. For comparison, signal at ‘common’ REs is shown, which include 10,000 regulatory elements with DNAse hypersensitivity across most or all cell types^20^ (Methods). Boxplots indicate area under the curve for the aggregate H3K27ac profile for each sample. Lower, middle, and upper hinges indicate 25^th^, 50^th^, and 75^th^ percentiles; whiskers extend to 1.5 x the inter-quartile ranges (IQR). Wilcoxon test two-sided *p*-values are indicated for comparison of healthy vs cancer samples.

**Extended Data Fig. 7:**
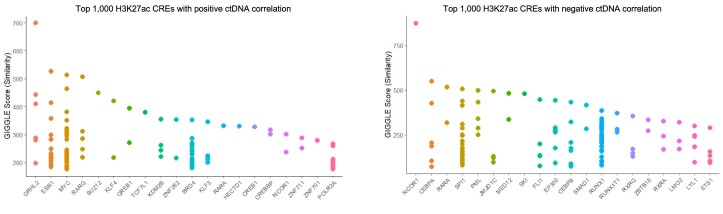
Transcription factor binding sites overlapping H3K27ac CREs. Overlap of the top 1,000 H3K27ac ctDNA correlated regions (CREs) with TF binding sites (TFBS) in cistromedb^28^. Giggle scores quantify the degree of overlap between CREs and TFBS as described^23,28^.

**Extended Data Fig. 8:**
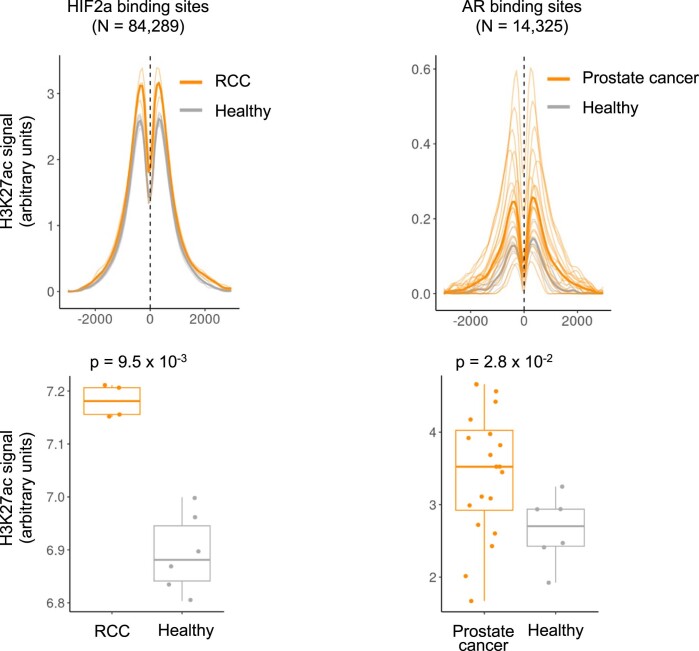
Aggregate H3K27ac cfChIP-seq signal at HIF2α binding sites in renal cell carcinoma (RCC) and at AR binding sites in prostate cancer. Healthy volunteer samples are shown for comparison. Boxplots indicate area under the curve for the aggregate H3K27ac profile for each sample. Lower, middle, and upper hinges indicate 25^th^, 50^th^, and 75^th^ percentiles; whiskers extend to 1.5 x the inter-quartile ranges (IQR). Wilcoxon test two-sided *p*-values are indicated for comparison of healthy vs cancer samples.

The ability to assess enhancer activity from plasma with H3K27ac provided distinct, clinically actionable insights into gene regulation compared with promoter profiling. Enhancer profiling from cancer plasma captured the activity of cancer REs that were defined independently in tumors using assay for transposase-accessible chromatin with sequencing (ATAC-seq)^14^ (Fig. 2a,b and Extended Data Fig. 6). Enhancer CREs were enriched for overlap with the binding sites of TFs that are protooncogenes, such as MYC, ER, EZH2, SUZ12 and BRD4 (Extended Data Fig. 7). Enhancer profiling from plasma allowed us to infer activity of therapeutically targetable TFs from plasma, including estrogen receptor (ER) in breast cancer plasma, androgen receptor (AR) in prostate cancer and HIF2α in renal cell carcinoma (RCC) (Extended Data Fig. 8). This functional readout of TF activity represents an advance from previous ctDNA assays and provides orthogonal information to TF gene promoter H3K4me3 levels. For example, the *ESR1* gene (encoding ER) is bivalently marked (H3K4me3^+^ and H3K27me3^+^) in ER^−^ breast cancer^15^. Accordingly, H3K4me3 at the *ESR1* promoter distinguished ER status only modestly compared to H3K27ac signal at a set of 27,840 REs that are activated in ER^+^ breast cancer^4^ (Fig. 2c,d).

**Extended Data Fig. 9:**
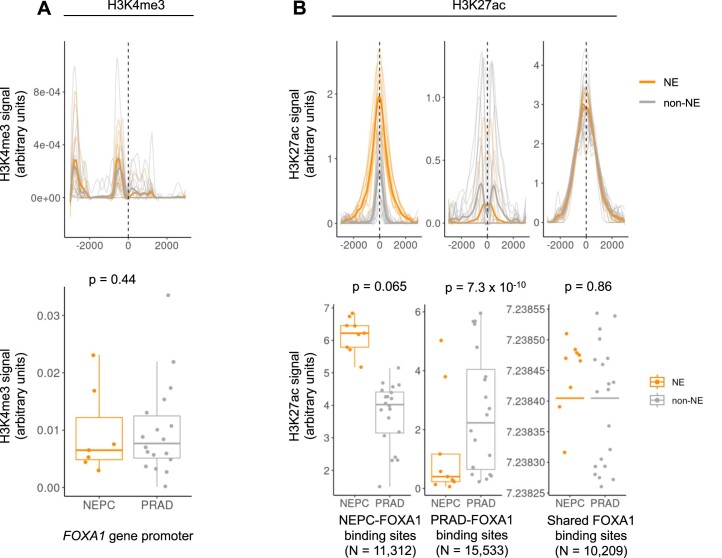
H3K27ac cfChIP-seq distinguishes prostate cancer subtype-specific FOXA1 binding sites. (**a**) H3K4me3 cfChIP-seq signal at the FOXA1 promoter in prostate adenocarcinoma (PRAD) vs. neuroendocrine prostate cancer (NEPC) for N = 25 biologically independent samples. (**b**) Aggregate H3K27ac cfChIP signal at Boxplots indicate aggregate signal at the indicated sites for the indicated epigenetic features for N = 29 biologically independent samples. NEPC-FOXA1 and PRAD-FOXA1 indicate FOXA1 binding sites that are preferentially bound in neuroendocrine prostate cancer (NEPC) compared to prostate adenocarcinoma (PRAD), as described previously^17^. Aggregate signal at differential FOXA1 binding sites for each sample is normalized to signal at shared FOXA1 binding sites that are common to NEPC and PRAD. Wilcoxon test two-sided *p*-values are indicated. Boxplots indicate area under the curve for the aggregate cfChIP-seq profile for each sample. Lower, middle, and upper hinges indicate 25^th^, 50^th^, and 75^th^ percentiles; whiskers extend to 1.5 x the inter-quartile ranges (IQR).

Enhancer profiling from plasma identified epigenetic drivers of treatment resistance. For instance, H3K27ac cell-free chromatin immunoprecipitation (cfChIP) detected activation of an enhancer of the *AR* gene that drives castration resistance in prostate cancer^16^ (Fig. 2e). Activation of the *AR* enhancer was not detectable from DNA methylation, because this locus is hypomethylated in benign and cancerous prostate tissue^16^, highlighting the utility of active enhancer profiling. Additionally, in plasma from patients with treatment-induced neuroendocrine differentiation (NE-diff) of prostate cancer, H3K27ac signal was elevated at binding sites for ASCL1 (a master TF driving NE-diff) and at NE-specific binding sites of FOXA1 (ref. ^17^) (Fig. 2f and Extended Data Fig. 9). Notably, genetically based assays are unable to detect this histologic transformation.

**Extended Data Fig. 10:**
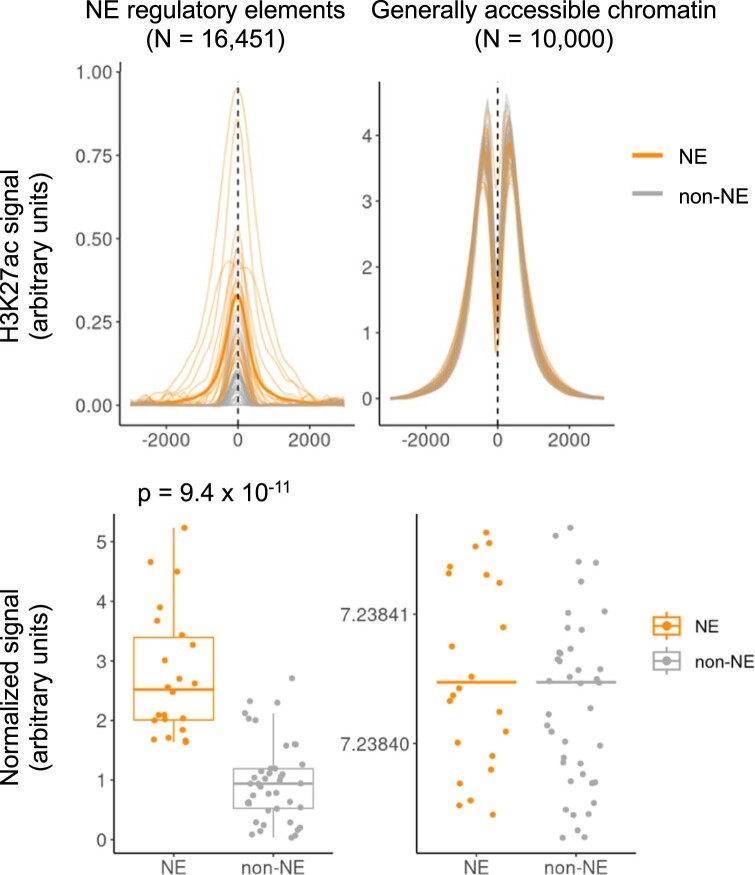
Aggregate H3K27ac cfChIP signal at neuroendocrine-enriched regulatory elements. Dark lines show the mean signal across all samples in the indicated class. ‘NE’ indicates samples with neuroendocrine differentiation (SCLC, NEPC, or Merkel cell carcinoma). Wilcoxon test two-sided *p*-value is indicated. Boxplots indicate area under the curve for the aggregate cfChIP-seq profile for each sample. Lower, middle, and upper hinges indicate 25^th^, 50^th^, and 75^th^ percentiles; whiskers extend to 1.5 x the inter-quartile ranges (IQR).

NE-diff is increasingly recognized as a mechanism of acquired resistance to targeted therapies in many cancers. Detection of NE-diff is clinically important because high-grade neuroendocrine tumors often respond to platinum-based chemotherapy, but spatial heterogeneity and sampling error make the pathologic diagnosis challenging. Therefore, we created a multi-cancer classifier of NE-diff from plasma, leveraging previous work that identified a common set of REs in neuroendocrine tumors across varying tissues of origin^18^. Aggregating plasma H3K27ac signal across neuroendocrine REs (*n* = 16,451) distinguished cancers with and without NE-diff (*n* = 22 and 42, respectively; area under the curve (AUC) = 0.94; Fig. 2g and Extended Data Fig. 10). Notably, this classifier was trained from published REs measured in cancer tissues, supporting its biological plausibility, and identified NE-diff in plasma from patients with prostate, lung, bladder and Merkel cell cancers.

Together, these results demonstrate that measuring gene regulation from patient plasma can identify clinically relevant disease phenotypes. This proof-of-concept study focused on metastatic cancer; further studies are needed to assess the utility of this approach in large prospective cohorts as well as its performance in early-stage disease and non-oncologic conditions. Another limitation of this approach is that it does not capture the spatial distribution of cell types and gene expression that can be assessed with tissue biopsy.

Because this assay requires only 1 ml of plasma from standard clinical collection tubes, it can be applied retrospectively to banked samples with clinical annotations, where sample volumes are often limiting. Because histone modifications are deposited and removed dynamically, they provide a real-time readout of gene regulation to complement DNA methylation, which tends to reflect cellular lineage^19^. This attribute should enable the in vivo study of acquired therapy resistance driven by epigenetic changes and allow longitudinal assessment of therapeutic targets whose expression changes with disease progression.

## Methods

### Study oversight and sample acquisition

This research complies with all relevant ethical regulations. Plasma samples were collected from various patient cohorts for this study as listed in Supplementary Table 1. Informed content was obtained in each case, and samples were de-identified. Plasma samples from the Dana-Farber Cancer Institute were collected under the following protocols approved by the Dana-Farber/Harvard Cancer Center (DF/HCC): 17-324 for patients with triple-negative breast cancer, 16-588 for patients with metastatic hormone receptor-positive breast cancer, 14-147 for patients with non-small cell lung cancer (NSCLC), 02-180 for patients with small cell lung cancer (SCLC), 05-042 for patients with melanoma, 10-417 for patients with glioma, 01-045 for patients with neuroendocrine prostate cancer (NEPC), 03-189 for patients with colorectal and esophageal cancers and 09-156 for patients with Merkel cell carcinoma. Patients had metastatic cancer unless otherwise noted.

Plasma samples from patients treated at the National Cancer Institute were collected under the following clinical trial protocols: hepatocellular carcinoma (11-C-0102), CRC (12-C-0187, 15-C-0021), ovarian cancer (12-C-0191), lung cancer (05-C-0049, 08-C-0078), prostate cancer (08-C-0074, 10-C-0062), RCC (02-C-0130) and thymic cancer (08-C-0033, 10-C-0077). All patients gave written informed consent in accordance with federal, state and institutional guidelines. The studies were conducted according to the Declaration of Helsinki and were approved by the National Cancer Institute Central Institutional Review Board (IRB).

Plasma samples from healthy individuals without a history of diabetes, cancer or major medical illnesses were obtained from the Mass General Brigham Biobank. Written informed consent was obtained from all healthy donors, and sample collection was approved by the Brigham and Women’s Hospital IRB (2009P002312), following ethical regulations.

Individual-level data, including sex and patient age, were not collected, except for PSA levels for patients with prostate cancer. Sex and/or gender were not considered in the study design.

Blood samples were collected in the tubes containing K2 EDTA (BD Biosciences, 366643), and plasma extraction was performed within 1–6 h of the blood draw. Whole blood was centrifuged for 10 min at 1,500*g* and 4 °C. Supernatant was transferred to a new conical tube and subjected to another centrifugation (for 10 min at 1,500*g* and 4 °C). After adding protease inhibitor (Roche, 11873580001), the extracted plasma was aliquoted, flash frozen and stored at −80 °C until use.

### cfChIP-seq assay

Next, 1 μg of antibody was coupled with 10 μl of protein A (Invitrogen, 10002D) and 10 μl of protein G (Invitrogen, 10004D) for at least 6 h at 4 °C with rotation in 0.5% BSA (Jackson Immunology, 001-000-161) in PBS (Gibco, 14190250), followed by blocking with 1% BSA in PBS for 1 h at 4 °C with rotation. The following antibodies were used, all at a dilution of 1 μg per 900 μl: H3K4me3, Thermo Fisher Scientific, PA5-27029; H3K27ac, Abcam, ab4729; and panAc, Active Motif, 39139.

Thawed plasma was centrifuged at 3,000*g* for 15 min at 4 °C. The supernatant was pre-cleared with the magnetic beads with 20 μl of protein A and 20 μl of protein G for 2 h at 4 °C. Then, the pre-cleared and conditioned plasma was subjected to antibody-coupled magnetic beads overnight with rotation at 4 °C. The reclaimed magnetic beads were washed with 1 ml of each washing buffer twice. Three washing buffers were used in the following order: low-salt washing buffer (0.1% SDS, 1% Triton X-100, 2 mM EDTA, 150 mM NaCl, 20 mM Tris-HCl, pH 7.5), high-salt washing buffer (0.1% SDS, 1% Triton X-100, 2 mM EDTA, 500 mM NaCl, 20 mM Tris-HCl, pH 7.5) and LiCl washing buffer (250 mM LiCl, 1% NP-40, 1% Na deoxycholate, 1 mM EDTA, 10 mM Tris-HCl, pH 7.5). Subsequently, the beads were rinsed with TE buffer (Thermo Fisher Scientific, BP2473500) and resuspended and incubated in 100 μl of DNA extraction buffer containing 0.1 M NaHCO_3_, 1% SDS and 0.6 mg ml^−1^ Proteinase K (Qiagen, 19131) and 0.4 mg ml^−1^ RNaseA (Thermo Fisher Scientific, 12091021) for 10 min at 37 °C, for 1 h at 50 °C and for 90 min at 65 °C. DNA was purified through phenol extraction (Invitrogen, 15593031), and ethanol precipitation was performed with 3 M NaOAc (Ambion, AM9740) and glycogen (Ambion, AM9510). cfChIP-seq libraries were prepared with ThruPLEX DNA-Seq Kit (Takara Bio, R400675) following the manufacturer’s instructions. After library amplification, the DNA was purified by AMPure XP (Beckman Coulter, A63880). The size distribution of the purified libraries was examined using Agilent 2100 Bioanalyzer with a high-sensitivity DNA Chip (Agilent, 5067-4626). The library was submitted for 150-bp paired-end sequencing on an Illumina NovaSeq 6000 system (Novogene).

### Low-pass whole-genome sequencing

cfDNA was extracted from plasma supernatant after cfChIP by QIAmp Circulating Nucleic Acid Kit (Qiagen, 55114) following the manufacturer’s instructions, and its concentration was measured with a Qubit fluorometer. Ninety percent of the extracted cfDNA was used for the subsequent Cell-free methylated DNA immunoprecipitation (cfMeDIP) library preparation (see below), and the remaining 10% of cfDNA was used for the library preparation by KAPA Hyper Prep Kit (Kapa Biosystems, KK8500) according to the manufacturer’s protocol. The final amplification cycle number was determined by additional qPCR using KAPA SYBR FAST qPCR Kits (Kapa Biosystems, KK4600). The library DNA profile was investigated using a TapeStation system and sequenced on an lllumina NovaSeq 6000 system with 150-bp paired-end sequencing (Novogene).

### cfMeDIP and high-throughput sequencing assay

cfMeDIP and high-throughput sequencing (cfMeDIP-seq) was performed as described^2^. In brief, cfDNA libraries were prepared using the KAPA HyperPrep Kit (Kapa Biosystems) according to the manufacturer’s protocol. We performed end-repair, A-tailing and ligation of NEBNext adaptors (NEBNext Multiplex Oligos for Illumina kit, New England Biolabs (NEB), E7645L). Libraries were digested using the USER enzyme (NEB, M5505S). λ DNA, consisting of unmethylated and in vitro methylated DNA, was added to prepared libraries to achieve a total amount of 100 ng of DNA. Methylated and unmethylated *Arabidopsis thaliana* DNA (Diagenode, C02040019) was added for quality control. DNA was heat denatured at 95 °C for 10 min and then immediately snap cooled on ice for 10 min. Then, 5-mC antibody from the MagMeDIP Kit (Diagenode, C02010021) was subjected to each sample following the manufacturer’s protocol at a dilution of 1:100. Samples were purified using the iPure Kit v2 (Diagenode, C03010015). Immunoprecipitation quality was confirmed using qPCR to measure recovery of the spiked-in *Arabidopsis thaliana* methylated versus unmethylated DNA. The DNA libraries were assessed for quality using a TapeStation system (Agilent Technologies) and sequenced on an lllumina NovaSeq 6000 system with 150-bp paired-end sequencing (Novogene).

### Sequence data processing

cfChIP-seq/cfMeDIP-seq reads were aligned to the hg19 human genome build using Burrows–Wheeler Aligner version 0.7.1740. Non-uniquely mapping and redundant reads were discarded. MACS version 2.1.1.2014061641 was used for ChIP-seq peak calling with a *q* value (false discovery rate (FDR)) threshold of 0.01. Fragment locations were converted to BED files using BEDTools (version 2.29.2) bamtobed with the -bedpe flag set. For analyses involving overlap with genomic regions, fragments were imported as GRanges objects and collapsed to 1 bp at the center of the fragment location to ensure that a fragment can map to only one site.

ChIP-seq data quality was evaluated by several measures, including the number of total unique fragments and total peaks. The distribution of fragment sizes was assessed to verify the expected bi-modal or tri-modal distribution characteristic of cfDNA.

To assess immunoprecipitation specificity, we calculated an on-target to off-target enrichment ratio. The enrichment ratio was calculated separately for promoter (H3K4me3) and promoter/enhancer (H3K27ac/panH3Ac) marks and reflects the density of fragments mapping to sites that are marked in most cell types (on-target sites) compared to sites that are not marked in any cell type (off-target sites). On-target sites were identified from the 18-state chromHMM maps generated by EpiMap (https://egg2.wustl.edu/roadmap/web_portal/chr_state_learning.html#exp_18state; accessed on 4 October 2021). For H3K27ac/panH3Ac on-target sites, we selected 200-bp windows with any of the following ‘active’ chromatin states in more than 50% of tissues in EpiMap: 1_TssA, 3_TssFlnkU, 8_EnhG2 and 9_EnhA1. On-target sites for H3K4me3 were selected similarly but using the following chromatin states: 1_TssA, 2_TssFlnk, 3_TssFlnkU, 4_TssFlnkD, 8_EnhG2 and 14_TssBiv. Off-target sites were defined as 200-bp windows that lacked the on-target annotations in all of 129 samples used to generate chromatin state maps in EpiMap. On-target and off-target windows were merged and retained if the merged windows spanned 1,000 bp or more. Off-target regions within 10,000 bp of on-target regions were excluded.

Unless otherwise specified, we included samples in downstream analysis if the on-target to off-target enrichment ratio was >10 and the product of the unique fragment number and enrichment ratio was >4 × 10^7^.

### Identification of CREs

CREs were identified where cfChIP-seq or cfMeDIP-seq signal correlated with low-pass whole-genome sequencing (LP-WGS)-based ctDNA estimates. We identified CREs separately for each data type (H3K4me3, H3K27ac, pan-H3ac and MeDIP) and for each cancer type where there were ≥5 samples with ctDNA estimates >0.03. We excluded samples with ichorCNA estimates ≤0.03, because the algorithm is benchmarked down to this ctDNA content^21^. For each analysis, peaks from all samples were merged to generate a union set of peaks. Unique fragments overlapping each peak were counted to form a count matrix with peaks versus samples. Counts were normalized to the summed counts across common REs that are expected to be active across most tissue types. These common REs were defined as the 10,000 sites with DNAse hypersensitivity across the largest number of samples in ref. ^20^. At each site, the Spearman correlation was tested between normalized signal and ctDNA content. We reported the top 1,000 sites by significance for each analysis as well as all CREs with FDR-adjusted *q* < 0.05.

CREs were assessed for overlap with gene features and CpG islands using annotatr and ChIPSeeker^22^. Normalized cfChIP-seq read counts at specific genomic loci were visualized with IGV version 2.8.243. The GREAT tool48 (version 3.0) was used to assess for enrichment of Gene Ontology (GO) and Molecular Signatures Database perturbation annotations among genes near CREs. The cistromedb toolkit (http://dbtoolkit.cistrome.org/) was used to compare H3K27ac CREs with peaks from a large database of uniformly analyzed published ChIP-seq data (quantified as a ‘GIGGLE score’)^23^. Published TFs and histone modification ChIP-seq datasets were ranked by similarity to the querry cfChIP-seq dataset based on the top 1,000 peaks by enrichment in each published dataset. Before cistromedb toolkit analysis, ChIP-seq peaks were mapped from hg19 to hg38 using the UCSC liftover tool (https://genome.ucsc.edu/cgi-bin/hgLiftOver).

### ctDNA estimation

ctDNA estimates were obtained from LP-WGS data using ichorCNA^21^ with default settings. For samples that lacked LP-WGS, we used signal at CREs to estimate ctDNA content. We fit a linear model to predict LP-WGS-based tumor fraction estimates (T) given the signal at CREs that were negatively and positively correlated at CRE (C_pos_ and C_neg_, respectively):

$$\frac{T+0.01}{1-(T+0.01)}\, \sim {\log }_{2}\frac{{C}_{{pos}}}{{C}_{{neg}}}$$

Where possible, we used CREs identified on a given cancer type to estimate ctDNA in samples of that type. In cases where there were too few samples to estimate cancer-type specific CREs, we used CREs identified using all cancer types. Estimates were scaled such that the mean estimate for healthy plasma, which was not used for CRE identification, was 0. In cases with LP-WGS-based ctDNA estimates, we report these rather than CRE-based estimates. Supplementary Table 1 lists the source of ctDNA estimates for each sample.

### Assessment of gene promoter activity based on H3K4me3

To estimate gene promoter activity, we quantified H3K4me3 near promoters. First, we merged all H3K4me3 cfChIP-seq peak calls into a single GRanges object and reduced them to non-overlapping intervals using the reduce() function. We removed peaks in high-noise regions (https://github.com/Boyle-Lab/Blacklist/blob/master/lists/hg19-blacklist.v2.bed.gz). For each peak, we normalized H3K4me3 fragment counts to the aggregate counts in a given sample across a set of 10,000 regions with DNAse hypersensitivity across most cell types^20^, as described above. We assigned peaks to genes based on proximity to transcriptional start sites in the annotation package TxDb.Hsapiens.UCSC.hg19.knownGene.

The genes highlighted in this manuscript were curated based on their clinical use in IHC for identifying cancer types or predictive markers. To assess whether our estimation of promoter signal was applicable beyond this set of genes, we also took a systematic approach for selecting genes in the classifier described below.

### Cancer classification based on promoter signal

Logistic regression with ℓ_2_-norm regularization was used to train biologically grounded and robust classifiers based on promoter H3K4me3 at lineage-enriched genes from the Human Protein Atlas (HPA)^24^ using scikit-learn^25^. The classifier considered 12,664 genes that were annotated as ‘tissue enriched’ or ‘tissue enhanced’ as well as ‘Not detected in immune cells’ in the HPA database. We employed a tenfold cross-validation technique to assess the performance of the predictive models. Within each fold, we fine-tuned the model’s hyperparameters using a threefold cross-validation approach, specifically on the training samples. Our objective was to optimize the algorithm parameters to maximize the AUC. To measure the model’s performance, we exclusively used the test samples and reported the average AUC values over the ten folds. We classified all cancer plasma samples versus healthy samples and classified cancer type for the three most abundant types in our cohort (prostate, lung and colorectal cancer).

### Enhancer signal quantification at transcription factor binding sites

We inferred RE activity at transcription factor binding sites (TFBSs) based on H3K27ac at these sites. This approach builds upon previous work that measured signals of nucleosome depletion in cfDNA at phenotype-defining REs^4,5^. Samples were included in this analysis only if they had >4 × 10^6^ unique fragments and, except for healthy volunteer plasma, estimated ctDNA content >0.03. We first filtered out sites with peaks present in plasma types that were not considered for a given analysis and that had zero estimated ctDNA content, to exclude sites with high background signal from nucleosomes that do not originate from cancer. MACS2 peak calls for TFBS were obtained, filtered to remove sites of width >4 kb and then resized to a 3-kb interval centered on the original peak. Peaks were separated into 40-bp windows, and fragment counts were aggregated across a given window for all peaks to obtain aggregate profiles for a sample. We performed two normalization steps. First, to account for variation in background signal across samples, we performed a ‘shoulder normalization’ step. We considered the region between [−3,000, −2,800] bp and [2,800, 3000] bp around the center of each TFBS and aggregated counts at these sites for each sample. This value was subtracted from the aggregate counts to set the ‘shoulder’ of peaks to zero. Second, we normalized signal in each bin to the aggregated signal at the common 10,000 DNAse hypersensitivity sites as described above.

### Correlation of cfChIP signal with expression

We measured correlation of promoter H3K4me3 cfChIP-seq signal in a representative healthy volunteer plasma sample (HP030642) with RNA sequencing (RNA-seq)-based gene expression measurements. For gene expression, we used transcripts per million (TPM) annotations for whole blood from GTEx, because most nucleosomes in healthy individuals derive form hematopoietic cells. To aggregate signal across multiple genes, we first ranked all genes by expression in whole blood and then created metagenes containing promoter cfChIP-seq signal from approximately 10 genes of similar expression levels. Signal was measured as fragment counts between 500 bp upstream and 1,500 bp downstream of the gene transcriptional start site. This analysis was also performed using cfMeDIP-seq from the same individual for comparison with H3K4me3.

### Detection of NE-diff

We classified samples by the activity of REs associated with NE-diff, as assessed by H3K27ac cfChIP-seq signal at these REs. Our feature set was a group of 16,098 sites with chromatin accessibility that is consistently higher in neuroendocrine tumors of multiple lineages compared to adenocarcinomas^18^. These sites were obtained from the original set of 16,571 sites by filtering out sites with peaks present in healthy volunteer H3K27ac cfChIP-seq profiles. We measured H3K27ac cfChIP-seq signal at these sites as described above for ‘enhancer signal quantification of TFBS’. The aggregated and normalized signal at these sites was used as an input to the classifier. Classifier performance was assessed by measuring the area under the receiver operating characteristic (ROC) curve.

### Detection of Merkel cell polyomavirus DNA

Reads that failed initial alignment (unmapped reads) were mapped to an hg19 assembly that contained viral sequences^26^. The resulting alignment files were then filtered where only properly paired reads with high mapping quality (mapq ≥30) and a minimal number of mismatches ((NM) ≤1) were kept, and duplicate reads were removed. Viral read counts were then quantified using BEDTools multicov^27^, and TPM was calculated.

### Statistics and reproducibility

Sample sizes were determined by sample availability. No statistical method was used to predetermine sample size, but numbers of samples exceeded those in previous studies^1–8^. All data generated for this study are included and reported here. For most analyses, we imposed quality cutoffs based on unique fragment counts and enrichment. Unless otherwise specified, we included samples in downstream analyses if the on-target to off-target fragment enrichment ratio was >10 and the product of the unique fragment number and enrichment ratio was >4 × 10^7^. The experiments were not randomized. The investigators were not blinded to allocation during experiments and outcome assessment.

### Reporting summary

Further information on research design is available in the Nature Portfolio Reporting Summary linked to this article.

## Online content

Any methods, additional references, Nature Portfolio reporting summaries, source data, extended data, supplementary information, acknowledgements, peer review information; details of author contributions and competing interests; and statements of data and code availability are available at 10.1038/s41591-023-02605-z.

### Supplementary information

Reporting Summary

Supplementary Table Excel spreadsheet containing Supplementary Tables 1 and 2.

## Data Availability

BED files containing genomic alignments of all sequenced fragments as well as ChIP-seq peak locations are available through GEO under accession number GSE243474. Due to privacy restrictions regarding genomic data, raw sequencing data can be shared upon reasonable request under a data use agreement. Requests should be directed to the corresponding author at freedman@broadinstitute.org and should receive a response within 2 weeks. The following public datasets were used: DNAse hypersensitivity sites (https://zenodo.org/record/3838751/files/DHS_Index_and_Vocabulary_hg19_WM20190703.txt.gz), TCGA ATAC-seq peak calls (https://api.gdc.cancer.gov/data/116ebba2-d284-485b-9121-faf73ce0a4ec; lifted over to hg19 from hg38), Human Protein Atlas database annotations (https://www.proteinatlas.org/download/proteinatlas.tsv.zip) and Encode list of high-noise regions for exclusion from ChIP-seq analysis (https://github.com/Boyle-Lab/Blacklist/blob/master/lists/hg19-blacklist.v2.bed.gz).

## Code availability

Scripts to reproduce analyses from this study are available at https://github.com/Baca-Lab/cfchip_manuscript.
