# Author Correction: Liquid biopsy epigenomic profiling for cancer subtyping

**DOI:** 10.1038/s41591-023-02735-4

**Published:** 2023-12-04

**Authors:** Sylvan C. Baca, Ji-Heui Seo, Matthew P. Davidsohn, Brad Fortunato, Karl Semaan, Shahabbedin Sotudian, Gitanjali Lakshminarayanan, Miklos Diossy, Xintao Qiu, Talal El Zarif, Hunter Savignano, John Canniff, Ikenna Madueke, Renee Maria Saliby, Ziwei Zhang, Rong Li, Yijia Jiang, Len Taing, Mark Awad, Cindy H. Chau, James A. DeCaprio, William D. Figg, Tim F. Greten, Aaron N. Hata, F. Stephen Hodi, Melissa E. Hughes, Keith L. Ligon, Nancy Lin, Kimmie Ng, Matthew G. Oser, Catherine Meador, Heather A. Parsons, Mark M. Pomerantz, Arun Rajan, Jerome Ritz, Manisha Thakuria, Sara M. Tolaney, Patrick Y. Wen, Henry Long, Jacob E. Berchuck, Zoltan Szallasi, Toni K. Choueiri, Matthew L. Freedman

**Affiliations:** 1 Department of Medical Oncology, Dana-Farber Cancer Institute, Boston, MA USA; 2 Center for Functional Cancer Epigenetics, Dana-Farber Cancer Institute, Boston, MA USA; 3 Eli and Edythe L. Broad Institute, Cambridge, MA USA; 4 Computational Health Informatics Program, Boston Children’s Hospital, Boston, MA USA; 5 Lowe Center for Thoracic Oncology, Dana-Farber Cancer Institute, Harvard Medical School, Boston, MA USA; 6 Molecular Pharmacology Section, Genitourinary Malignancies Branch, Center for Cancer Research, National Cancer Institute, National Institutes of Health, Bethesda, MD USA; 7 Department of Medicine, Brigham and Women’s Hospital, Harvard Medical School, Boston, MA USA; 8 Liver Cancer Program, Center for Cancer Research, National Cancer Institute, National Institutes of Health, Bethesda, MD USA; 9 Massachusetts General Hospital Cancer Center, Boston, MA USA; 10 Department of Medicine, Massachusetts General Hospital and Harvard Medical School, Boston, MA USA; 11 Department of Pathology, Dana-Farber Cancer Institute, Boston, MA USA; 12 Thoracic and Gastrointestinal Malignancies Branch, Center for Cancer Research, National Cancer Institute, National Institute of Health, Bethesda, MD USA; 13 Department of Dermatology, Brigham and Women’s Hospital, Harvard Medical School, Boston, MA USA; 14 Center for Cutaneous Oncology, Dana-Farber/Brigham and Women’s Cancer Center, Boston, MA USA; 15 Center for Neuro-Oncology, Dana-Farber Cancer Institute, Boston, MA USA; 16 Department of Neurology, Brigham and Women’s Hospital and Harvard Medical School, Boston, MA USA; 17 Danish Cancer Institute, Copenhagen, Denmark; 18 Department of Bioinformatics and Department of Pathology, Forensic and Insurance Medicine, Semmelweis University, Budapest, Hungary

**Keywords:** Tumour biomarkers, Predictive markers, Biomarkers, Translational research, Epigenomics

Correction to: *Nature Medicine* 10.1038/s41591-023-02605-z, published online 21 October 2023.

This article was originally published under a standard Springer Nature license (© The Author(s), under exclusive licence to Springer Nature America, Inc.); it is now available as an open-access paper under a Creative Commons Attribution 4.0 International license, © The Author(s). The error has been corrected in the HTML and PDF versions of the article.

