# Supplementary material for: Liquid biopsy epigenomic profiling for cancer subtyping
Source: Nat Med. 2023 Oct 21;29(11):2737–41. doi: 10.1038/s41591-023-02605-z (PMC10695830; doi:10.1038/s41591-023-02605-z)
Supplement: Supplementary file 1 — Reporting Summary [file 41591_2023_2605_MOESM1_ESM.pdf]

nature portfolio

Corresponding author(s): Matthew Freedman MD

Last updated by author(s): Sep 18, 2023

Reporting Summary

Nature Portfolio wishes to improve the reproducibility of the work that we publish. This form provides structure for consistency and transparency in reporting. For further information on Nature Portfolio policies, see our Editorial Policies and the Editorial Policy Checklist.

Statistics

For all statistical analyses, confirm that the following items are present in the figure legend, table legend, main text, or Methods section.

n/a	Confirmed	
☐	☒	The exact sample size (*n*) for each experimental group/condition, given as a discrete number and unit of measurement
☒	☐	A statement on whether measurements were taken from distinct samples or whether the same sample was measured repeatedly
☐	☒	The statistical test(s) used AND whether they are one- or two-sided<br>*Only common tests should be described solely by name; describe more complex techniques in the Methods section.*
☒	☐	A description of all covariates tested
☒	☐	A description of any assumptions or corrections, such as tests of normality and adjustment for multiple comparisons
☐	☒	A full description of the statistical parameters including central tendency (e.g. means) or other basic estimates (e.g. regression coefficient) AND variation (e.g. standard deviation) or associated estimates of uncertainty (e.g. confidence intervals)
☐	☒	For null hypothesis testing, the test statistic (e.g. *F*, *t*, *r*) with confidence intervals, effect sizes, degrees of freedom and *P* value noted<br>*Give P values as exact values whenever suitable.*
☒	☐	For Bayesian analysis, information on the choice of priors and Markov chain Monte Carlo settings
☒	☐	For hierarchical and complex designs, identification of the appropriate level for tests and full reporting of outcomes
☐	☒	Estimates of effect sizes (e.g. Cohen's *d*, Pearson's *r*), indicating how they were calculated

Our web collection on statistics for biologists contains articles on many of the points above.

Software and code

Policy information about availability of computer code

Data collection	No software was used for data collection.
Data analysis	Code to reproduce analyses from this study is available at https://github.com/Baca-Lab/cfchip_manuscript. The following versions of software were used: RStudio 2023.03.1+446, R version 4.2.2 (2022-10-31), Burrows-Wheeler Aligner (BWA) version 0.7.1740, MACS v2.1.1.2014061641, bedtools v2.29.2.

For manuscripts utilizing custom algorithms or software that are central to the research but not yet described in published literature, software must be made available to editors and reviewers. We strongly encourage code deposition in a community repository (e.g. GitHub). See the Nature Portfolio guidelines for submitting code & software for further information.

Data

Policy information about availability of data

All manuscripts must include a data availability statement. This statement should provide the following information, where applicable:

- Accession codes, unique identifiers, or web links for publicly available datasets
- A description of any restrictions on data availability
- For clinical datasets or third party data, please ensure that the statement adheres to our policy

Bed files containing genomic alignments of all sequencing reads are available at Zenodo via the following links: https://zenodo.org/record/8353657, https://zenodo.org/record/8353863, and https://zenodo.org/record/8355970. ChIP-seq peak calls in bed format are available at https://zenodo.org/record/8356068. Due

to privacy restrictions regarding genomic data, raw sequencing data can be shared upon request under a data use agreement. Requests should be directed to the corresponding author at freedman@broadinstitute.org and should receive a response within two weeks.

The following public data sets were used: DNAse hypersensitivity sites (https://zenodo.org/record/3838751/files/DHS_Index_and_Vocabulary_hg19_WM20190703.txt.gz), TCGA ATAC-seq peak calls (https://api.gdc.cancer.gov/data/116ebba2-d284-485b-9121-faf73ce0a4ec; lifted over to hg19 from hg38), Human Protein Atlas database annotations (https://www.proteinatlas.org/download/proteinatlas.tsv.zip), Encode list of high-noise regions for exclusion from ChIP-seq analysis (https://github.com/Boyle-Lab/Blacklist/blob/master/lists/hg19-blacklist.v2.bed.gz).

Research involving human participants, their data, or biological material

Policy information about studies with human participants or human data. See also policy information about sex, gender (identity/presentation), and sexual orientation and race, ethnicity and racism.

Reporting on sex and gender	No sex-based or gender-based analyses were performed in this study as these data were not collected for most samples
Reporting on race, ethnicity, or other socially relevant groupings	No such groupings were used in this study
Population characteristics	Participants were adult (>= years of age) patients treated for advanced cancer at tertiary academic medical centers (the Dana-Farber Cancer Institute, Massachusetts General Hospital, or the National Cancer Institute) or cancer-free patients seen in primary care clinics at Mass General Hospital or Brigham and Women's Hospital. Individual-level and population-level data, including age, were not collected for this study.
Recruitment	Samples were obtained from patients treated for advanced cancers. Therefore, the extensibility of these results to patients with early stage cancers remains to be explored.
Ethics oversight	Plasma samples from the Dana-Farber Cancer Institute were collected under the following protocols approved by the Dana-Farber/Harvard Cancer Center (DF/HCC): 17-324 for patients with triple-negative breast cancer, 16-588 for patients with metastatic hormone receptor positive breast cancer, 14-147 for patients with NSCLC, 02-180 for patients with SCLC, 05-042 for patients with melanoma, 10-417 for patients with glioma, 01-045 for patients with NEPC, 03-189 for patients with colorectal and esophageal cancers, 09-156 for patients with Merkel cell carcinoma.<br><br>Plasma samples from patients treated at the National Cancer Institute were collected under the following clinical trial protocols: hepatocellular carcinoma (11-C-0102), colorectal cancer (12-C-0187, 15-C-0021), ovarian cancer (12-C-0191), lung cancer (05-C-0049, 08-C-0078), prostate cancer (08-C-0074, 10-C-0062), RCC (02-C-0130), and thymic cancer (08-C-0033, 10-C-0077). All the patients gave written informed consent in accordance with federal, state, and institutional guidelines. The studies were conducted according to the Declaration of Helsinki and were approved by the National Cancer Institute Central Institutional Review Board.<br><br>Plasma samples from healthy individuals without a history of diabetes, cancer, or major medical illnesses were obtained from the Mass General Brigham Biobank. Written informed consent was obtained from all healthy donors, and sample collection was approved by the Brigham and Women's Hospital IRB 2009P002312, following ethical regulations.

Note that full information on the approval of the study protocol must also be provided in the manuscript.

Field-specific reporting

Please select the one below that is the best fit for your research. If you are not sure, read the appropriate sections before making your selection.

☒ Life sciences ☐ Behavioural & social sciences ☐ Ecological, evolutionary & environmental sciences

For a reference copy of the document with all sections, see nature.com/documents/nr-reporting-summary-flat.pdf

Life sciences study design

All studies must disclose on these points even when the disclosure is negative.

Sample size	No sample size calculation was performed. Sample size was determined by sample availability. Where available, we aimed to sample 10 or more samples for each epigenetic mark for each cancer type as this approximate sample size has proven sufficient for identifying distinguishing epigenetic features of cancer subtypes in prior work (eg PMID: 36681680)
Data exclusions	All data generated for this study are included and reported here. For most analyses, we imposed quality cutoffs based on unique fragment counts and enrichment. Unless otherwise specified, we included samples in downstream analysis if the on-target to off-target enrichment ratio was > 10 and the product of the unique fragment number and enrichment ratio was > 4 x 10^7
Replication	Reproducibility was confirmed by generating high-quality cfChIP data on plasma samples collected at different centers. Experiments were performed by different operators at different times over the course of approximately 2 years. Similar data quality was observed across these conditions. ~12% of H3K27ac cfChIP-seq samples and ~ 26% of H3K4me3 were run multiple times.

Randomization	Randomization was not relevant to this study because it was performed on retrospectively collected samples
Blinding	Blinding was not relevant to this study because this study did not involve an intervention or prospective classification of samples/patients to different groups

Reporting for specific materials, systems and methods

We require information from authors about some types of materials, experimental systems and methods used in many studies. Here, indicate whether each material, system or method listed is relevant to your study. If you are not sure if a list item applies to your research, read the appropriate section before selecting a response.

Materials & experimental systems

n/a	Involved in the study
☐	☒ Antibodies
☒	☐ Eukaryotic cell lines
☒	☐ Palaeontology and archaeology
☒	☐ Animals and other organisms
☒	☐ Clinical data
☒	☐ Dual use research of concern
☒	☐ Plants

Methods

n/a	Involved in the study
☐	☒ ChIP-seq
☒	☐ Flow cytometry
☒	☐ MRI-based neuroimaging

Antibodies

Antibodies used	H3K4me3: Thermo Fisher # PA5-27029 (dilution 1ug/900uL)<br>H3K27ac: Abcam # ab4729 (dilution 1ug/900uL)<br>panAc : Active Motif # 39139 (dilution 1ug/900uL)<br>MeDIP : Diagenode # C02010021 (dilution 1:100)
Validation	Validation data and publications are listed on the manufacturers websites here:<br>https://www.thermofisher.com/antibody/product/H3K4me3-Antibody-Polyclonal/PA5-27029<br>https://www.abcam.com/products/primary-antibodies/histone-h3-acetyl-k27-antibody-chip-grade-ab4729.html<br>https://www.activemotif.com/catalog/details/39139/histone-h3ac-pan-acetyl-antibody-pab-1<br>https://www.diagenode.com/en/p/magmedip-kit-x48-48-rxns

Plants

Seed stocks	*Report on the source of all seed stocks or other plant material used. If applicable, state the seed stock centre and catalogue number. If plant specimens were collected from the field, describe the collection location, date and sampling procedures.*
Novel plant genotypes	*Describe the methods by which all novel plant genotypes were produced. This includes those generated by transgenic approaches, gene editing, chemical/radiation-based mutagenesis and hybridization. For transgenic lines, describe the transformation method, the number of independent lines analyzed and the generation upon which experiments were performed. For gene-edited lines, describe the editor used, the endogenous sequence targeted for editing, the targeting guide RNA sequence (if applicable) and how the editor was applied.*
Authentication	*Describe any authentication procedures for each seed stock used or novel genotype generated. Describe any experiments used to assess the effect of a mutation and, where applicable, how potential secondary effects (e.g. second site T-DNA insertions, mosiacism, off-target gene editing) were examined.*

ChIP-seq

Data deposition

☒ Confirm that both raw and final processed data have been deposited in a public database such as GEO.

☒ Confirm that you have deposited or provided access to graph files (e.g. BED files) for the called peaks.

Data access links<br>*May remain private before publication.*	Bed files containing genomic alignments of all sequencing reads are available at Zenodo via the following links: https://zenodo.org/record/8353657, https://zenodo.org/record/8353863, and https://zenodo.org/record/8355970. ChIP-seq peak calls in bed format are available at https://zenodo.org/record/8356068.
Files in database submission	The files are listed in Table S1 and omitted here for conciseness as there are > 2,000

Genome browser session (e.g. UCSC)

No longer applicable.

Methodology

Replicates	Replicates were not used in this study.
Sequencing depth	150bp paired-end sequencing was performed on the Illumina platform. The number of reads is indicated in Table S1 (median ~46 million paired end reads).
Antibodies	The following antibodies were used: H3K4me3, Thermo Fisher # PA5-27029; H3K27ac, Abcam # ab4729; panAc, Active Motif # 39139; MeDIP, Diagenode # C02010021.
Peak calling parameters	Narrow peaks were called on deduplicated bam files using the following command: macs2 callpeak --SPMR -B -q 0.01 --keep-dup 1 -g hs -f BAMPE --extsize 146 --nomodel -t {treat.bam} -c {input.bam}.
Data quality	Data quality were assessed by several means, including peak number, number of unique fragments, and on-/off-target enrichment ratio, as described in the methods.
Software	Code to reproduce analyses from this study is available at https://github.com/Baca-Lab/cfchip_manuscript.
